# Antimicrobial peptide LL-37 and recombinant human mannose-binding lectin express distinct age- and pathogen-specific antimicrobial activity in human newborn cord blood *in vitro*

**DOI:** 10.12688/f1000research.14736.1

**Published:** 2018-05-21

**Authors:** Annette Scheid, Ning Li, Carleen Jeffers, Francesco Borriello, Sweta Joshi, Al Ozonoff, Matthew Pettengill, Ofer Levy

**Affiliations:** 1 Department of Pediatric Newborn Medicine, Brigham and Women's University Hospital, Harvard Medical School, Boston, Massachusetts, USA; 2 Precision Vaccines Program, Division of Infectious Diseases, Boston Children's Hospital, Boston, Massachusetts, USA; 3 Harvard Medical School, Boston, Massachusetts, USA; 4 Department of Translational Medical Sciences, Center for Basic and Clinical Immunology Research (CISI), University of Naples Federico II, Naples, Italy; 5 WAO Center of Excellence, Naples, Italy; 6 Center for Patient Safety and Quality Research, Boston Children's Hospital, Boston, Massachusetts, USA; 7 Thomas Jefferson University, Philadelphia, Pennsylvania, USA; 8 Broad Institute of MIT & Harvard, Cambridge, Massachusetts, USA

**Keywords:** Newborn, Preterm, Cord Blood, Antimicrobial Activity, LL-37, Mannose Binding Lectin

## Abstract

**Background:** There is a need to prevent and treat infection in newborns. One approach is administration of antimicrobial proteins and peptides (APPs) such as LL-37, a membrane-active cathelicidin antimicrobial peptide, and mannose-binding lectin (MBL), a pattern-recognition protein that binds to microbial surface polysaccharides resulting in opsonization and complement activation. Low plasma/serum levels of LL-37 and of MBL have been correlated with infection and exogenous administration of these agents may enhance host defense.

**Methods:** The antimicrobial activity of LL-37 (15 µg/ml) or rMBL (0.5, 2 and 10 µg/ml) was tested in hirudin-anticoagulated preterm and term human cord blood (N = 12–14) against *Staphylococcus aureus* (SA) USA 300 (2x10 ^4^ CFU/ml), *Staphylococcus epidermis* (SE) 1457 (2x10 ^4 ^CFU/ml) and *Candida albicans* (CA) SC5314 (1x10 ^4 ^CFU/ml). After incubation (1, 45, or 180 min), CFUs were enumerated by plating blood onto agar plates. Supernatants were collected for measurement of MBL via ELISA.

**Results:** Preterm cord blood demonstrated impaired endogenous killing capacity against SA and SE compared to term blood. Addition of LL-37 strongly enhanced antimicrobial/antifungal activity vs SA, SE and CA in term blood and SE and CA in preterm blood. By contrast, rMBL showed modest fungistatic activity vs CA in a sub-analysis of term newborns with high basal MBL levels. Baseline MBL levels varied within preterm and term cohorts with no correlation to gestational age. In summary, exogenous LL-37 demonstrated significant antimicrobial activity against SA, SE and CA in term and SE and CA in preterm human blood tested *in vitro*. rMBL demonstrated modest antifungal activity in term cord blood of individuals with high baseline MBL levels.

**Conclusions:** To the extent that our *in vitro* results predict the effects of APPs *in vivo*, development of APPs for prevention and treatment of infection should take into account host age as well as the target pathogen.

## Introduction

Neonatal sepsis is a major contributor to neonatal morbidity and mortality; consequently, efforts to ease the burden of this disease are crucial ^1^. Sepsis reflects an infection-induced systemic inflammatory response syndrome ^2^. Early-onset sepsis (EOS) is most commonly differentiated from late-onset sepsis (LOS) by the onset occurring before or after the first 72 h of life, respectively ^3^. Of note, both the clinical features and pathophysiology of sepsis varies markedly by age, such that adult, pediatric and neonatal sepsis criteria are distinct ^1^. While screening and prophylaxis for Group B *Streptococcus* has reduced rates of EOS (i.e., within the first 3 days of life), LOS in the preterm infant has increased in frequency as a higher number of premature infants have survived, resulting in invasive procedures and prolonged hospital stays, as well as increased pathogen exposure ^3, 4^. LOS considerably lengthens the infant’s hospital stay, and is associated with long-term neurodevelopmental complications and a high risk of mortality ^1^. Risk of LOS is inversely related to birth weight and gestational age (GA); as such, preterm and very low birth weight infants are at a higher risk of infection ^5^. Accordingly, there is a need to reduce and mitigate neonatal LOS.

One approach to reducing and mitigating LOS in high-risk newborns is the use of immunomodulatory strategies. Among these, a promising area for investigation are antimicrobial proteins and peptides (APPs) ^6^. For example, administration of oral lactoferrin to preterm newborns reduces the risk of sepsis and necrotizing enterocolitis ^7^. In the present study we focused on the potential utility of two APPs with distinct modes of action: (a) the α-helical LL-37 cationic cathelicidin ^8^ is a broad spectrum membrane-active antimicrobial peptide that induces microbial lysis, blocks endotoxin activity, synergizes with other host defense systems, ^9^ and modulates inflammatory responses ^9, 10^; and (b) mannose-binding lectin (MBL), a host pattern recognition receptor that recognizes and binds to sugar moieties on the surface of bacteria and fungi, enhances opsonophagocytosis, and forms complexes with MBL-associated serine proteases that trigger complement activation ^11^. Indeed, relatively low plasma LL-37 or MBL concentrations are associated with a higher risk of infection ^9, 12^. Deficiencies in LL-37 or MBL levels can be genotypic ^13, 14^, such as genetic variants of exon 1 on the human MBL gene ( *MBL2*), or phenotypic such as reduced expression of APPs in preterm plasma ^15, 16^. Some premature infants have a distinct immune system, and some may be MBL-deficient, as defined in prior neonatal studies by plasma/serum concentrations <700 ng/ml ^11, 17^. Accordingly, it has been hypothesized that the administration of recombinant MBL (rMBL) as a supplement to bolster the neonatal innate immune system could reduce the risk of LOS ^11^. However, to our knowledge, no published studies have examined addition of rMBL to human newborn blood.

To characterize the activity of LL-37 and rMBL in neonatal blood, we evaluated antimicrobial activity towards three pathogens commonly associated with LOS in newborns: (a) *Staphylococcus epidermidis* (SE), that accounts for 78% of cases of LOS due to coagulase-negative staphylococci, (b) *Staphylococcus aureus* (SA), a less common pathogen associated with a high rate of mortality; and *Candida albicans* (CA) the most common fungal pathogen associated with LOS. We also conducted a sub-analysis with respect to rMBL effects in term cord blood with low baseline levels vs those with high baseline MBL levels. We found that these agents exerted distinct antimicrobial activity that depended on both pathogen and age. Specifically, rMBL demonstrated modest fungistatic activity vs CA in term newborns with high basal MBL levels. By contrast, LL-37 demonstrated substantial antimicrobial activity that was generally greater in term (SA, SE and CA) than in preterm (SE and CA) blood tested *in vitro*. The antimicrobial activity of rMBL and LL-37 *in vitro* depends on three factors: the baseline endogenous level of APP, the pathogen identity and the age of the host, informing the translational development of these promising agents.

## Methods

### APPs

rMBL, provided by Shire (Lexington, MA), was expressed in HEK293 cells and purified by affinity chromatography on Glucosamine Sepharose 4FF and ion exchange chromatography on Source 30Q and diafiltration (100 kDa) from GE Healthcare Life Sciences (Pittsburg, PA, USA), including a Benzonase DNA removal step from MilliporeSigma (Billerica, MA) or similar and several microfiltration and nanofiltration steps for bioburden and adventitious virus elimination. rMBL was provided frozen, aliquoted at 10× assay concentration, and stored in single-use quantities to minimize freeze-thaw. LL-37 was purchased from AnaSpec, Inc. (Fremont, California); it was purchased in 1 mg vials, re-suspended in 1 ml distilled water and frozen in aliquots (stock concentration 1 mg/ml) at -80°C.

### Microbial pathogens

The anti-infective effect of rMBL and LL-37 was assessed in three pathogens: (a) SE strain 1457, a clinical isolate from a central catheter infection (kindly provided by Dr. Michael Otto, National Institute of Allergy & Infectious Diseases, National Institutes of Health, Rockville, MD), was cultured in trypticase soy broth (TSB), as previously described ^18^; (b) SA strain USA300, a strain of community-associated methicillin-resistant SA (kindly provided by Dr. William Nauseef, University of Iowa; Iowa City, IA) that was cultured in TSB; and (c) CA strain SC5314 ^19^, (kindly provided by Dr. Julia Koehler, Division of Infectious Diseases, Boston Children’s Hospital, Boston, MA), which was cultured in yeast extract-peptone-dextrose (YPD) broth.

### Cord blood collection

Cord blood was obtained from 30 human newborns: 22 term newborns ranging from 37 0/7 to 40 4/7 weeks GA and 8 preterm newborns ranging from 26 1/7 to 36 6/7 weeks GA. Cord blood samples were collected at The Brigham and Women’s Hospital (BWH) and Beth Israel Deaconess Medical Center (BI), both tertiary care centers for newborn delivery and postnatal care. De-identified newborn cord blood was collected immediately after Caesarian section or vaginal delivery of the placenta from a large umbilical vein and was anti-coagulated with pyrogen-free hirudin (Verum Diagnostica GmbH, Munich, Germany). Since the mechanism of action of MBL involves complement activation, we used Hirudin as an anticoagulant which does not impact complement activation. We did not use Heparin or EDTA as coagulants, as Heparin, is known to bind to complement and EDTA may inhibit complement activation. Inclusion criteria were either term or preterm gestational age; and birth via vaginal delivery or caesarian section. Sample collections included both male and female newborns. Exclusion criteria were maternal fever peripartum (>104°F/40°C) or seropositive status for human immunodeficiency virus.

Patient information concerning the collected cord blood samples was collected in a de-identified manner and hence maternal consent was waived by the local institutional review boards at The Brigham and Women’s Hospital (Protocol #:2000P000117/BWH) and the Beth Israel Deaconess Medical Center (Protocol #2011P-000118/BIDMC. The data associated with our study has been provided in an Excel-compatible format.

### Assay protocol

A total of 10–20 ml of term or preterm cord blood was collected in hirudin vacutainers at room temperature and processed within 4 h of collection. A total of 1 ml hirudinated blood was centrifuged and plasma collected and cryopreserved at -80°C for subsequent evaluation of MBL concentrations via ELISA (Hycult®biotech; Cat. No. HK323-01). Endogenous LL-37 levels were not determined. LL-37 was prepared at 10× assay concentration in 1× saline. A total of 15 µl negative control (saline), rMBL (500 ng/ml, 2000 ng/ml, or 10,000 ng/ml), or LL-37 reagents (1 mg of protein/ml) as well as 15 µl SA strain USA300 (2×10 ^4^/ml), SE strain 1457 (2×10 ^4^/ml) or CA (final concentration 1×10 ^4^ CFU/ml) in saline were added to 120 µl hirudin-anticoagulated preterm and term human cord blood and incubated at 37°C. At 1, 45, and 180 min, 10–20 µl of each replicate was spread on tryptic soy blood agar plates to quantify colony forming units (CFUs). Plate CFUs were counted 16–18 h after assay commencement for SA and SE, or at 48 h after assay commencement for CA using the Accu Count™ 1000, Automated Colony Counter (BioLogics, Inc.). This automated colony counter was carefully calibrated, and the assay designed to ensure colony counts <200 colonies per plate in order to facilitate reliable colony counts. Of note, the cord blood collection volumes obtained permitted incubation with all three pathogens in 20 of the term patient samples, incubation with SA and SE but not CA in one term sample, and incubation with SA only in one term sample.

### Statistical analyses and graphics

**Figure 1.**
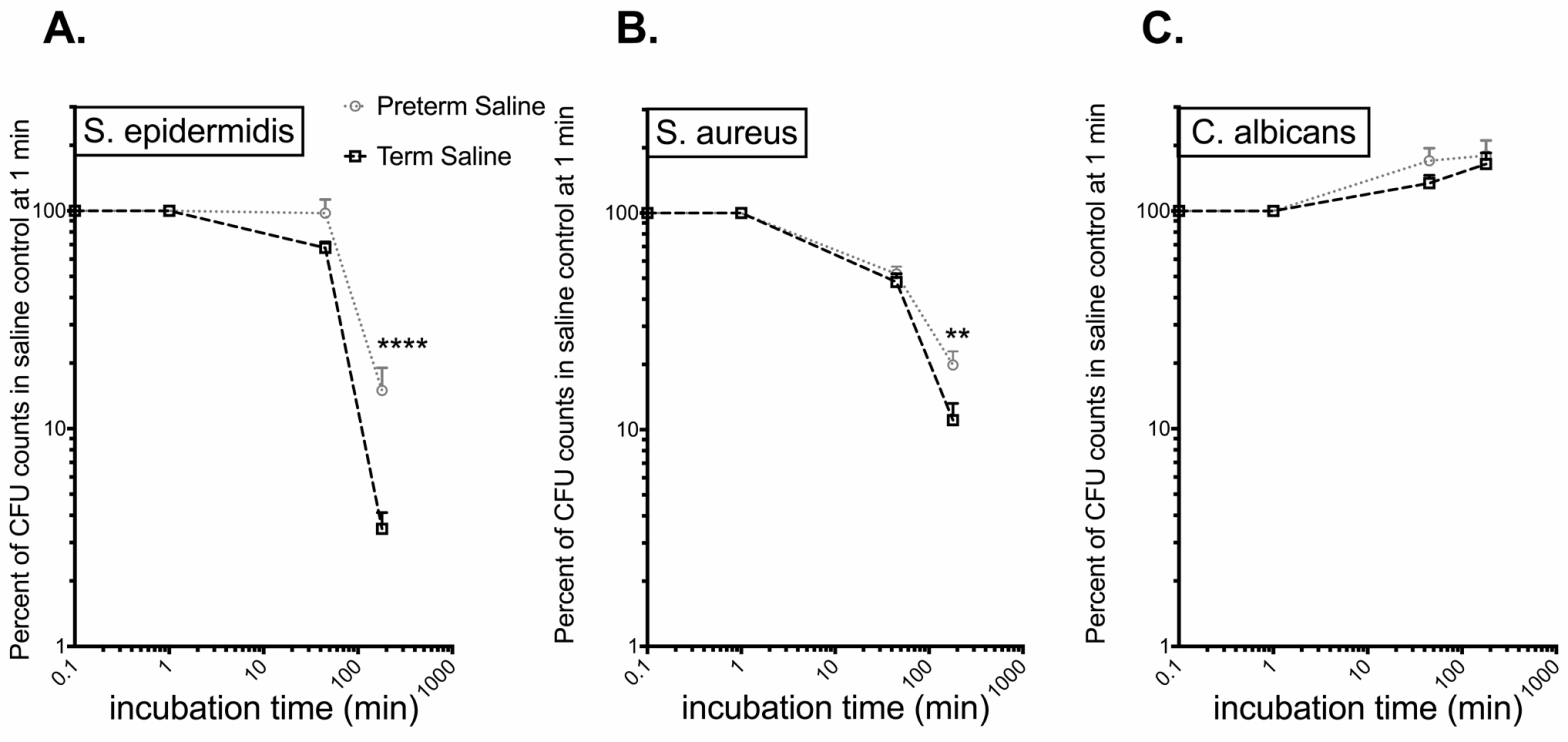
Preterm cord blood exhibits a lower killing capacity against *S. epidermidis* ( **A**) and *S. aureus* ( **B**) than term cord blood at 180 min. While the trend towards lower killing activity by preterm cord blood was observed for *C. albicans* ( **C**) at 45 min, the difference did not reach statistical significance. Killing capacity was measured at 1 min, 45 min and 180 min; the inoculum (SA and SE, 2x10 ^4^ CFU/ml; CA, 1x10 ^4^/ml) at time-point “0” is plotted at “0.1 min”. CFU counts are expressed in percent of CFUs detected at 1 min. Term, N = 20–22 (N = 20 for CA; N = 21 for SE; N = 22 for SA); preterm, N = 8. Statistical analysis was performed via two-way ANOVA with Sidak’s post hoc test. ****p<0.0001. **p<0.005. All graphs depict mean and error with SEM.

**Figure 2.**
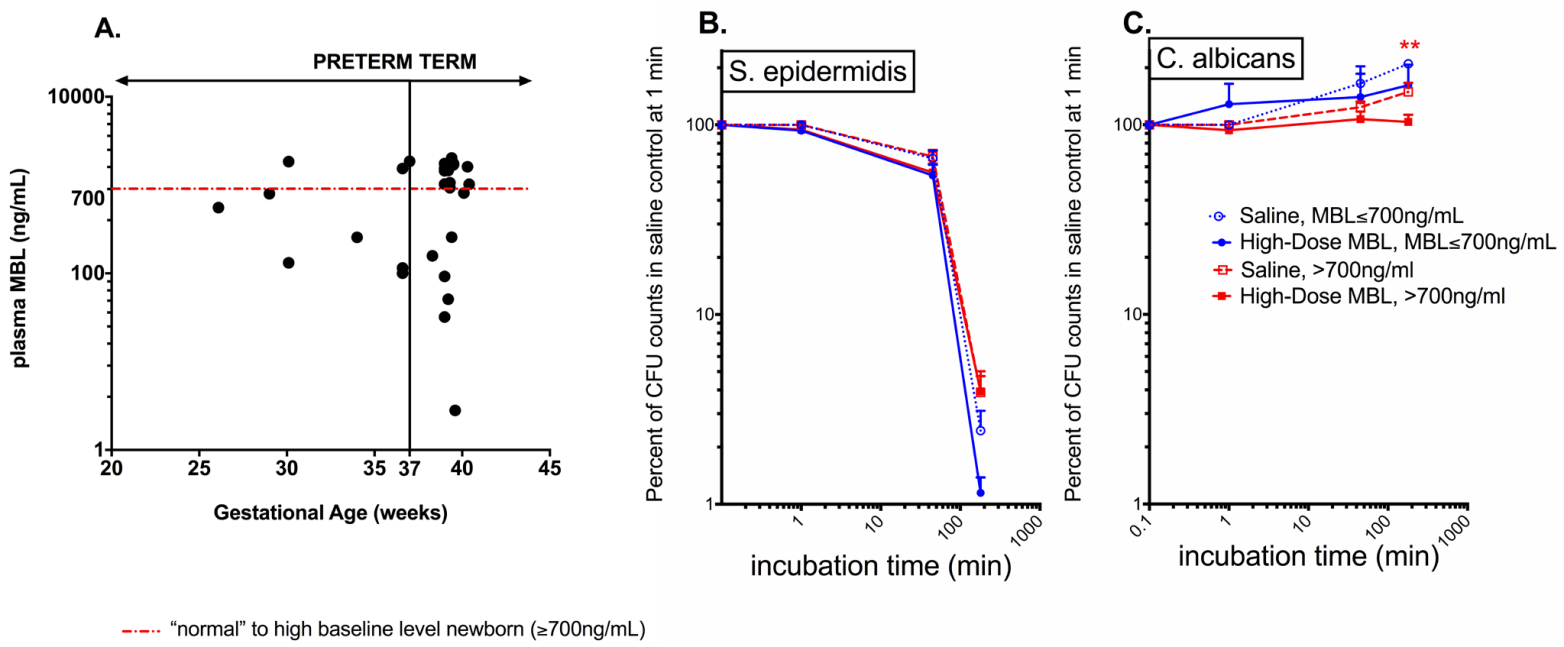
Exogenous MBL in term cord blood with high baseline MBL levels exerts modest antimicrobial activity towards *C. albicans* (CA) but not *S. epidermidis* (SE). ( **A**) Scattergram of plasma MBL levels as a function of gestational age. Baseline MBL levels that do not correlate with gestational age. ( **B**) No statistical significance in the antimicrobial effect against SE between MBL treatment vs saline in the low or high baseline MBL groups when analyzed separately. ( **C**) Enhanced antifungal effect of exogenous MBL towards CA in term newborns with high baseline MBL levels only. Statistical analysis was performed in panels ( **B**) and ( **C**) via repeated measure two-way ANOVA with Sidak’s post hoc test. ***p<0.005. All graphs depict mean and error with SEM. CFU counts was measured at 1 min, 45 min and 180 min, the inoculum (SA and SE, 2x10 ^4^ CFU/ml; CA, 1x10 ^4^ CFU/ml) at time-point “0” is plotted at “0.1 min”. A Spearman correlation was performed for panel ( **A**), which demonstrates no correlation between gestational age and baseline MBL levels in our cohort (r=0.1834, p=0.3319). CFU counts in ( **B**) and ( **C**) are expressed in percent of CFUs detected in saline treated control blood obtained from the same individual at 1 min. ( **A**) Baseline MBL levels detected in a total of 30 samples (22 term, 8 preterm); ( **B**) SE: Low baseline MBL N = 6; High baseline MBL N = 15; ( **C**) CA: Low baseline MBL N = 5; High baseline MBL N = 15.

**Figure 3.**
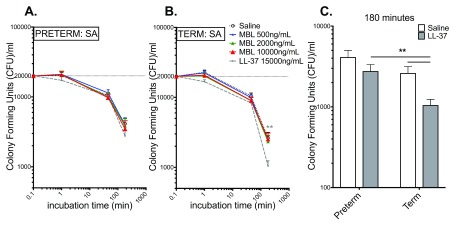
LL-37 significantly inhibits growth of *S. aureus* in term but not preterm cord blood relative to saline control at 180 min. Viability of SA as measured in CFU is plotted on the y-axis relative to the incubation time in panels ( **A**) and ( **B**). ( **A**) Neither LL-37 nor MBL inhibit growth of SA in preterm blood. ( **B**) LL-37 significantly inhibits SA growth in term blood at 180 min. ( **C**) Summary of LL-37 effects on SA growth in term and preterm cord blood at 180 min, demonstrating that the inhibitory effect of LL-37 is more pronounced in term than in preterm cord blood. The inoculum (2×10 ^4 ^CFU/ml) at time-point “0” is plotted at 0.1 min. Term, N = 20–22; preterm, N = 8. **p<0.005. Statistical analysis employed two-way ANOVA with Dunnett’s ( **A**, **B**) or Sidak’s ( **C**) post hoc test. All graphs depict mean and error with SEM.

**Figure 4.**
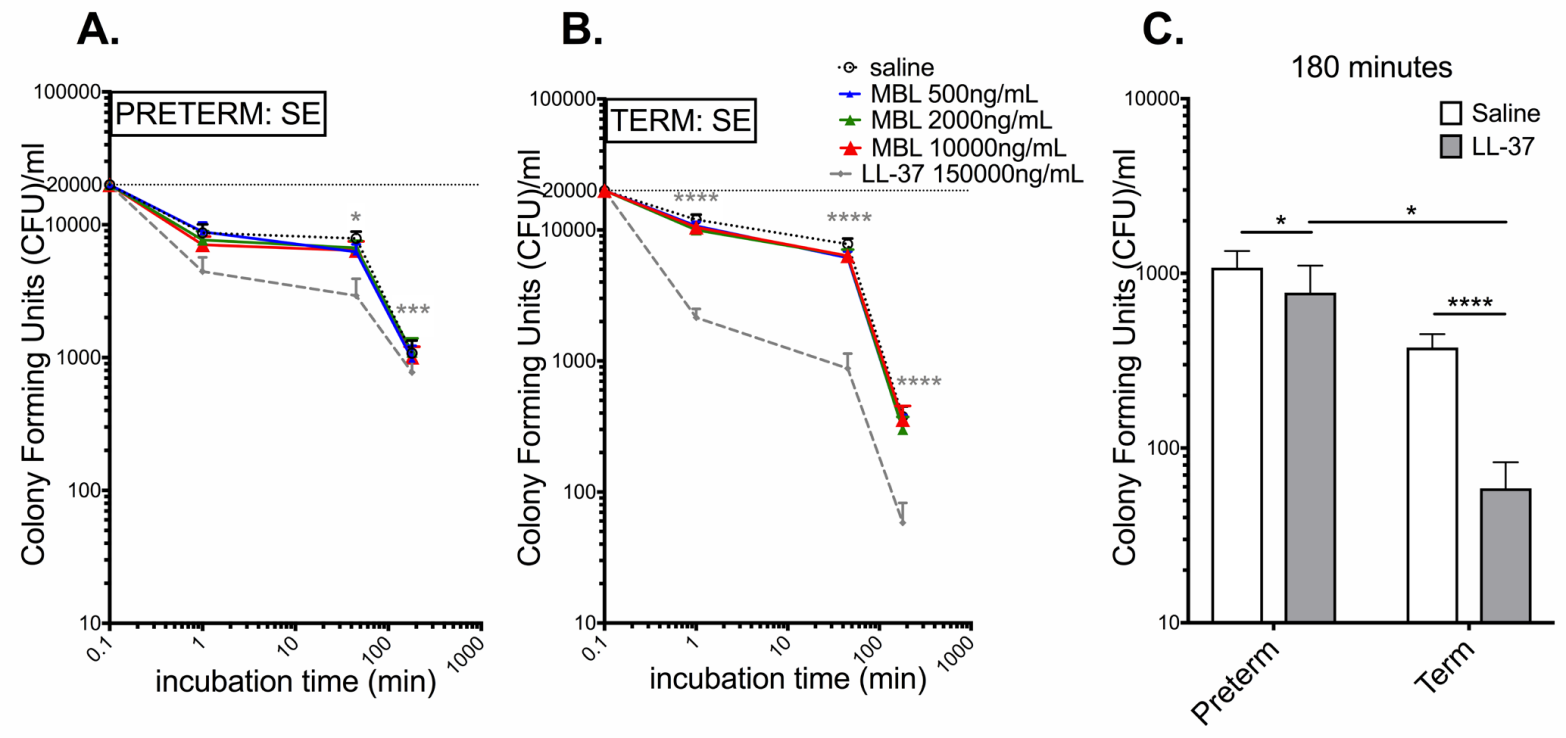
LL-37, but not MBL, exhibits antibacterial activity towards *S. epidermidis* (SE) in human preterm and term cord blood relative to saline control. Viability of SE as measured in CFU is plotted on the y-axis relative to the incubation time in panels ( **A**) and ( **B**). ( **A**) LL-37 but not MBL inhibits growth of SE in preterm blood at 45 min and 180 min. ( **B**) LL-37 significantly inhibits SE growth in term blood at 1 min, 45 min and 180 min. ( **C**) Summary of LL-37 effects on SE growth in term and preterm cord blood at 180 min, demonstrating that the inhibitory effect of LL-37 is stronger in term than in preterm cord blood. The inoculum (2x10 ^4^ CFU/ml) at time-point “0” is plotted at 0.1 min. Term N = 21–22 (N = 20 for CA; N = 21 for SE; N = 22 for SA); preterm N = 8. Statistical analyses employed two-way ANOVA with Dunnett’s ( **A, B**) or Sidak’s ( **C**) post hoc test. *p<0.05, ***p<0.0005, ****p<0.0005. All graphs depict mean and error ± SEM.

**Figure 5.**
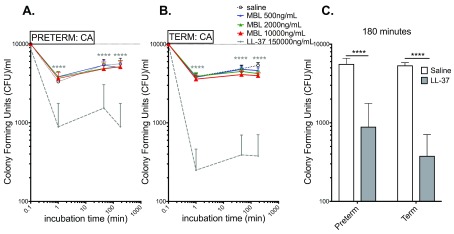
LL-37, but not MBL, demonstrates antimicrobial activity towards *C. albicans* in preterm and term cord blood relative to saline control. Viability of CA as measured in CFU is plotted on the y-axis relative to the incubation time in panels ( **A**) and ( **B**). ( **A**) LL-37 but not MBL inhibits growth of CA in preterm blood at 1 min, 45 min and 180 min. ( **B**) Significant LL-37 inhibition of SE growth in term blood at 1 min, 45 min and at 180 min. ( **C**) Summary of LL-37 effects on SE growth in term and preterm cord blood at 180 min, demonstrating an equally pronounced inhibitory effect of LL-37 on CA growth in term and preterm cord blood. The inoculum (1x10 ^4^ CFU/ml) at time-point “0” is plotted at 0.1 min. Term N = 20; preterm N = 8. Statistical analysis employed two-way ANOVA with Dunnett’s ( **A**, **B**) or Sidak’s ( **C**) post hoc test. **** p<0.0005. All graphs depict mean and error with SEM.

Data were analyzed and graphed using Prism for MacIntosh v. 7.0 (GraphPad Software, Inc.). Tests used for statistical comparisons are indicated in the figure legends. P values <0.05 were considered significant. Statistical analysis was performed via two-way ANOVA with either a Sidak’s post hoc test ( Figure 1, Figure 2 panels (B) and (C), Figure 3 panel (C), Figure 4 panel (C) and Figure 5 panel (C) or Dunnett’s post hoc test ( Figure 3 panels (A) and (B), Figure 4 panels (A) and (B), and Figure 5 panels (A) and (B). In Figure 2 panel (A), Spearman’s correlation was performed.

## Results

### Preterm cord blood demonstrates lesser killing activity against SA and SE than term cord blood

Overall, bacterial viability decreased over time in our whole-blood assay ( Figure 1). In accordance with the known deficiency of antimicrobial mechanisms in preterm infants, preterm cord blood demonstrated significantly lower killing capacity against SE ( Figure 1A) or SA ( Figure 1B) than term cord blood at 180 min. The viability of CA increased modestly over time in both preterm and term cord blood, with no significant differences observed between age groups ( Figure 1C).

### Exogenous rMBL in term cord blood exerts modest antimicrobial activity towards CA in term cord blood with high basal MBL levels. Basal MBL levels did not appear to be GA-dependent

Individuals were stratified into high vs low baseline plasma MBL values, using a threshold of 700 ng/ml, in keeping with past studies ^11^. Baseline MBL concentrations within both preterm and term groups varied broadly ( Figure 2A). GA and baseline MBL level were not significantly correlated (Spearman r = 0.18, p = 0.33). This suggests that MBL concentrations did not vary by GA, in agreement with the results of other groups ^20^. In our term cohort, exogenous rMBL, when added to high baseline MBL cord blood, showed a modest fungistatic effect against CA when compared with saline treated control high baseline MBL term cord blood at 180 min ( Figure 2C). By contrast, exogenous rMBL demonstrated no bactericidal effect against SE in low or high baseline MBL term cord blood ( Figure 2B). With respect to SA, there was no significant effect of high-dose rMBL addition to term cord blood with low or high baseline MBL levels (data not shown).

### LL-37, but not rMBL, significantly inhibits growth of SA in term, but not preterm, cord blood

Figure 3 demonstrates the effects of addition of LL-37 as well as rMBL at three different concentrations on the growth of SA in preterm and term cord blood. In preterm cord blood, the addition of neither rMBL nor LL-37 inhibited the growth of SA relative to the saline control ( Figure 3A). By contrast, in term cord blood, LL-37 significantly decreased SA growth at 180 min, whereas rMBL did not ( Figure 3B). The inhibitory effect of LL-37 on SA growth was more pronounced in term cord blood than in preterm cord blood ( Figure 3C).

### LL-37, but not rMBL, exhibits antibacterial activity towards S. epidermidis in human preterm and term cord blood

As demonstrated in Figure 4, LL-37 demonstrated a pronounced inhibitory effect on SE growth in both preterm ( Figure 4A) and term cord blood ( Figure 4B) at 45 and 180 min. In term cord blood this effect was evident at 1 min incubation. rMBL showed no bactericidal effect against SE in preterm ( Figure 4A) or term ( Figure 4B) cord blood. When comparing the bactericidal effect of LL-37 at 180 min in term cord blood to preterm cord blood, the effect in term cord blood was more pronounced ( Figure 4C).

### LL-37, but not rMBL, demonstrates antimicrobial activity towards CA in preterm and term cord blood relative to saline control

Figure 5 demonstrates the significant growth inhibitory effect of LL-37 on CA growth in preterm ( Figure 5A) and term cord blood ( Figure 5B) relative to saline control at all three time points measured. rMBL demonstrated no inhibitory effect on CA growth in preterm or term cord blood. The inhibitory effect of LL-37 on growth of CA at 180 min was as significant in preterm cord blood as it was in term cord blood ( Figure 5C).

The complete raw data for the study, organized per figure Click here for additional data file. Copyright: © 2018 Scheid A et al. 2018 Data associated with the article are available under the terms of the Creative Commons Zero "No rights reserved" data waiver (CC0 1.0 Public domain dedication).

## Discussion

In this study we have, to our knowledge for the first time, characterized the antimicrobial activity of exogenous LL-37 and rMBL when added to human preterm and term cord blood *in vitro*. While some studies suggest that relatively low serum MBL or LL-37 levels are associated with a risk of specific infections ^9, 12, 22– 25^, to our knowledge, including PubMed search as of date 2/24/18 using the term “LL-37” and “cord blood”, or “mannose binding lectin” and “cord blood”, none have measured the activity of these APPs when added to preterm or term newborn blood.

Of note, preterm cord blood demonstrated a lower killing capacity against SA and SE than term cord blood. To our knowledge, this has not been demonstrated previously. As killing may be both extracellular and/or intracellular, this impairment in killing may reflect known deficits in plasma APP content with GA ^16^ and/or impaired preterm neutrophil function, such as reduced chemotaxis and chemokinesis ^26^.

In our cohort of newborns, MBL levels were markedly variable among both preterm and term cord blood samples and thus did not seem to correlate with GA ( Figure 2A), consistent with studies demonstrating that cord blood MBL levels most closely reflect MBL genotype distribution rather than GA ^20^.

In our study, MBL, at the concentrations tested in hirudinated whole blood, did not inhibit growth of SA, SE or CA in term or preterm cord blood. MBL in a sub-analysis of basal MBL levels, did exert modest fungistatic activity against CA in term newborn blood.

LL-37 demonstrated significant antimicrobial and antifungal activity towards SE, SA and CA in term cord blood. It also demonstrated strong antimicrobial effects against SE and antifungal effects against CA in preterm cord blood. LL-37 generally exerted lesser antibacterial activity in preterm than in term blood, suggesting that it may act together with other host defense components that increase with GA. Of note, amongst other APPs, LL-37 levels are expressed in human breast milk, which demonstrated bacterial growth inhibitory effects towards both SA and SE, with activity towards SE increasing with the postnatal age of the breast milk expression ^27^. LL-37 has previously been demonstrated to be a potent antimicrobial in adult peripheral blood ^28^.

Our study featured several strengths, including the use of a species- and GA-specific human whole blood assay system that is: (a) relatively physiological, (b) has been predictive of APP activity *in vivo* ^29^, and (c) enables blood samples from the same individual to be assayed in both control and treatment conditions, including testing across a time range to characterize kinetic effects, thereby enhancing statistical power via paired analyses. Our study also has several limitations including: (a) relatively greater number of cord blood samples from term study participants (N = 22) than from preterm participants (N = 8), limiting the power to detect age-specific differences; (b) an absence of measurement of endogenous LL-37 levels due to sample and logistical limitations; and (c) limitations of the whole blood assay which, although it is often predictive, does not perfectly model *in vivo* conditions, including blood flow and endothelial interactions.

In conclusion, rMBL exhibited very modest fungistatic properties when added to term cord blood with high baseline MBL levels. By contrast, LL-37 inhibited the growth of SA, SE and CA in term cord blood, and SE and CA in preterm cord blood. To the extent that our *in vitro* system is relevant *in vivo*, LL-37 and its congeners ^30^, such as immunoglobulin-based constructs that enhance half-life, may be promising agents to prevent and/or treat neonatal sepsis. Further translational studies of LL-37 designed to take into account both the pathogen identity and GA of the target population are warranted.

## Data availability

The data referenced by this article are under copyright with the following copyright statement: Copyright: © 2018 Scheid A et al.

Data associated with the article are available under the terms of the Creative Commons Zero "No rights reserved" data waiver (CC0 1.0 Public domain dedication). 

**Dataset 1. The complete raw data for the study, organized per figure.** DOI: 10.5256/f1000research.14736.d203317 ^21^.
