# Supplementary material for: Antimicrobial peptide LL-37 and recombinant human mannose-binding lectin express distinct age- and pathogen-specific antimicrobial activity in human newborn cord blood in vitro
Source: F1000Res. 2018 May 21;7:616. [Version 1] doi: 10.12688/f1000research.14736.1 (PMC6143923; doi:10.12688/f1000research.14736.1)
Supplement: Peer review history

# Peer review: Referee response for version 1

**DOI:** 10.5256/f1000research.16033.r36281

**Published:** 2018-09-14

**Authors:** Jerrold Weiss

**Affiliations:** 1 AffiliationInflammation Program, University of Iowa, Iowa City, IA, 52242-1182, USA

The broader goals (seeking ways to enhance possibly limiting host defense capacities of pre-term and term newborns) are important, the experimental approach for these target samples (though not more generally) is novel, some (but not all) of the limits of this approach recognized, and the microbial targets and exogenously added host-derived (via recombinant form) reasonable.  Of note, for experiments of this nature, the general reproducibility of the observations are striking. The description of the assay protocol should provide a more complete description of the "physical format" of the incubations (e.g., vessels used; type of sample shaking (or not)) that could impact, especially, the participation of the cellular elements of the blood samples that are likely important in the handling and outcome of these infections *in vivo*.

 The main "weakness" of this study is that the findings seem not surprising and not leading to an obvious next chapter unless other test host agents come to mind and/or become available. The conclusions of the authors, I think, reflect a similar view. The demonstration of lower killing capacity of preterm vs term cord blood against the two staphylococcal species tested is a new finding that may be important but deserves a more thorough characterization.  It is not surprising that this difference could also impact the potential efficacy of the added agents as shown in Figs. 3-5. How that works would be of interest and potential value. In the end, the choice of MBL and LL-37 -- considering their limited effects in very high test doses -- may not have been optimal.

I have read this submission. I believe that I have an appropriate level of expertise to confirm that it is of an acceptable scientific standard.

## References

[1] SjoestrandU: [General anesthesia and bronchoscopy]. *Ann Anesthesiol Fr*.1976;17(8) :871-7 12688

# Peer review: Referee response for version 1

**DOI:** 10.5256/f1000research.16033.r35932

**Published:** 2018-07-20

**Authors:** Cinzia Auriti

**Affiliations:** 1 Department of Medical and Surgical Neonatology, Bambino Gesù Children's Hospital , Rome, Italy

This is an extremely interesting research paper, well conducted from the methodological point of view and the statistical processing of data, well written and with well documented results.

 The idea of administering recombinant MBL to newborns with a MBL deficit is not new and could open many unconventional ways in treating neonatal infections. Therefore, studies that increase biological knowledge in this area are very relevant.

 The observation to be made is that the side effects of MBL are not yet known exactly. MBL, on the one hand, promotes the defense against infections, but on the other if in excess, could by itself generate tissue damage, thanks to induction activity of complement system. This problem is more evident in newborns, who have little biological ability to contain inflammatory up regulation. So until today we do not know if we can safely administer recombinant MBL to human neonates without side effects.

 That said, which could be cited in discussion, the study is experimental, of great interest because it adds important knowledge (type of bacteria, relationships with prematurity and baseline MBL levels) and in my opinion can be published.

I have read this submission. I believe that I have an appropriate level of expertise to confirm that it is of an acceptable scientific standard.

# Response

**Published:** 2018-07-20

**Authors:** Annette Scheid

**Affiliations:** Brigham and Women's Hospital, USA

We thank Dr. Auriti, a renown expert in neonatology as well as neonatal immunology and sepsis biology for her support in publishing our proposed article.
